# Reuse of Industrial and Agricultural Waste in the Fabrication of Geopolymeric Binders: Mechanical and Microstructural Behavior

**DOI:** 10.3390/ma14092089

**Published:** 2021-04-21

**Authors:** Jordi Payá, Lourdes Soriano, Alba Font, Maria Victoria Borrachero Rosado, Javier Alejandro Nande, Jose María Monzo Balbuena

**Affiliations:** ICITECH-GIQUIMA Grupo de Investigación en Química de los Materiales de Construcción, Instituto de Ciencia y Tecnología del Hormigón, Universitat Politècnica de València, 46022 València, Spain; jjpaya@cst.upv.es (J.P.); lousomar@upvnet.upv.es (L.S.); fontpeal@gmail.com (A.F.); javier.nande90@gmail.com (J.A.N.); jmmonzo@cst.upv.es (J.M.M.B.)

**Keywords:** fluid catalytic cracking residue, ceramic sanitary ware, rice husk ash, diatomaceous earth, waste, alkali activated materials, resource recovery

## Abstract

Resource recovery from waste is one of the most important ways to implement the so-called circular economy, and the use of alkali activated materials can become an alternative for traditional PC-based materials. These types of materials are based on waste resources involving a lower carbon footprint and present similar or high properties and good durability compared to that Portland cement (PC). This research work proposes using new waste generated in different types of industries. Four waste types were employed: fluid catalytic cracking residue (FCC) from the petrochemical industry; ceramic sanitary ware (CSW) from the construction industry; rice husk ash (RHA); diatomaceous waste from beer filtration (DB) (food industry). FCC and CSW were employed as precursor materials, and mixtures of both showed good properties of the obtained alkali activated materials generated with commercial products as activators (NaOH/waterglass). RHA and DB were herein used as an alternative silica source to prepare the alkaline activating solution. Mechanical behavior was studied by the compressive strength development of mortars. The corresponding pastes were characterized by X-ray diffraction, thermogravimetric analysis, and microscopy studies. The results were satisfactory, and demonstrated that employing these alternative activators from waste produces alkali activated materials with good mechanical properties, which were sometimes similar or even better than those obtained with commercial reagents.

## 1. Introduction

Sustainable Development Goals (SDGs) call to work towards prosperity while protecting the planet. The United Nations proposed 17 goals to transform the world. Goal 13 indicates the need to take urgent action to combat climate change and its impacts [1].

Global carbon dioxide (CO_2_) emissions have grown around 50% since 1990, and emissions have grown even more quickly between 2000 and 2010 than in the three previous decades. CO_2_ is one of the greenhouse gases responsible for the climate change [2].

The cement industry is responsible for about 8% of global CO_2_ emissions, of which 5% are caused by the carbonate decomposition process [3]. For this reason, all alternatives that can reduce cement use are welcome to reduce gas emission levels. Shi et al. reviewed the recent progress in low-carbon binder [4] by writing about the principal groups of low-carbon binders: alkali-activated cements and geopolymers, carbonate binders, belite-ye´elimite-ferrite binders. With alkali-activated cements and geopolymers, precursor materials in most cases are by-products or waste from other industries [5,6,7,8]. It is true, as Shi et al. [4] stated, that a critical point for employing this type of materials is the availability of these materials in specific locations. Additionally, the reactivity and the nature of the reaction products in alkali activated materials depends on the chemical composition of the precursor and activator, and the reaction extent [9].

In the last decade, researchers have investigated the use of waste to totally or partially replace commercial reagents in manufacturing geopolymers or alkali-activated cements [10,11,12,13]. The use of RHA as a silica source has been explored in different publications with diverse treatments to obtain the activator, and combining them with various precursors [1,2,3,4,5,6,7,8,9,10,11,12,13,14,15,16,17,18]. For example, Geraldo et al. [18] carried out a treatment of RHA with NaOH lasting 30 min at 90 °C with a magnetic stirrer. Kamseu et al. [16] introduced RHA into NaOH solution, and the mixture was ball-milled in a porcelain jar for 40 min.

Mellado et al. [19] studied the reducing CO_2_ emissions in mortars with FCC and RHA as silica sources. They concluded that the mortar with RHA presented a 50% reduction compared to the mortar prepared with Na_2_SiO_3_. Pasuello et al. [20] studied the life cycle impact assessment (LCA) with different categories. They concluded that the mortar with RHA could reduce impacts in six of the nine parameters assessed, and would lead to a reduction of more than 70% in the global warming potential (GWP) compared to the Portland cement (PC) mortar.

The use of diatomaceous earth (DE) as an activator is less known than RHA. The first reference was published by Mejía et al. [21], who compared the use of RHA and calcined spent DE from the brewing industry, using fly ash (FA) and metakaolin (MK) as precursors. They described the silica source dissolution process by NaOH: the mixture of both components was suspended in water and stirred for 24 h at room temperature. Bagci et al. [22] used natural DE and calcined the material at 400 °C, which was mixed with MK as a precursor. To dissolve the silica from DE, the authors mixed KOH, deionized water, and DE in a closed container, and the mixture was stirred overnight. Finally, Font et al. [23] employed dried diatomaceous earth that derived from the beer filtration process (DB) and used FCC as precursor. They dissolved the silica of the DB with NaOH in a thermal bottle for 24 h.

A review is presented of the current production data of all the waste types herein employed. Around 840,000 metric tons of FCC are produced worldwide per year [24]. The production of FCC in 2019 came to 423 tons (data provided by the BP refinery located in Grao de Castellón, Spain). In the present research work, the FCC from this refinery was used because it is the closest refinery to the university facilities. Ten refineries operate in Spain. It has been demonstrated that the diverse origins of catalysts do not affect their reactivity as pozzolan in cement matrices [25]. A study should be conducted to verify that there is no difference in alkali activated matrices either.

The other material employed in large quantities to produce alkali activated matrices is CSW. The products catalogued as CSW are diverse, and include bidets, cisterns, sinks, etc. The weight of such products is between 7.6 kg and 39.5 kg per piece [26]. Using the data published by Cuviella et al. [27] on the worldwide CSW production in 2014 (349.3 millions of pieces), and considering an average weight of 20 kg per piece, the CSW production in 2014 was around 7 million tons, and production in Europe was around 1.18 million tons. If we take into account that rejected pieces are around 5–7% [28], rejected CSW would be around 70,800 tons only in Europe. To this quantity we should add the pieces collected from demolition practices, for which no data are available.

Moaeyedi et al. [29] published a paper with RHA applications based on the data of the Food and Agricultural Organization of the United Nations (FAO). They published that the worldwide rice production in 2016 was 533 million tons. One ton of rice produces 0.2 of husk, and one ton of husk generates 220 kg of ash. Therefore, the potential amount of RHA produced in 2016 would have been some 23.45 million tons. Aprianti [30] published data about the world’s RHA production. The data for 2013 in Europe gave 0.1 million ton. Spain is the second largest rice producer in Europe behind Italy. According to data from the Spanish Ministry of Agriculture, Fisheries and Food [31], the RHA produced in 2015 came to 37,100 tons.

Finally, DB production was estimated from published data. Dessalew et al. [32] indicated for each liter of beer, around 17.14 grams of brewery spent diatomite sludge is produced, and the moisture of this sludge is 70%: around 5.14 grams of dry spent diatomite is produced for 1 liter of manufactured beer. This sludge needs to be treated, but is often dumped in landfills. In 2017, 3.6 billion liters of beer were produced in Spain [33]. According to the figures proposed by Dessalew et al. [32], the calculated quantity of spent diatomite produced in Spain would be around 1850 tons of DB.

The authors of the present paper studied the viability of manufacture alkali activated binders using local waste. The starting point for this research was a previous publication, in which a mixture of fluid catalytic cracking residue (FCC) and ceramic sanitary ware (CSW) was used as a precursor and commercial reagents as activators (NaOH and Na_2_SiO_3_) [34]. The influence of the replacement percentage of CSW by FCC, and also of the SiO_2_ concentration in the activator were herein studied. It was concluded that the addition of FCC improved the mechanical properties of CSW-based binders. The best results were obtained by the mortars with 50% FCC and using 7.28 mol of SiO_2_ per kg of water. However, the compressive strength value obtained with the mortar with 30% FCC and using 4.37 mol of SiO_2_ per kg of water was sufficient according to common construction use requirements. The novelty of the present research lies in employing alternative sources of silica to replace the use of waterglass for activating CSW and FCC as precursors. Selected alternative sources of silica are rice husk ash (RHA) and a diatomaceous earth deriving from the beer filtration process (DB).

The objective of the present research was to study the compressive strength and physico-chemical characteristics of the first alkali activated mixtures employing FCC and CSW as precursors and RHA and DB as alternative activators. Two types of curing conditions were tested to compare the reaction evolution at different temperatures. To support the behavior of the obtained materials, an analysis of the corresponding pastes was carried out.

## 2. Materials and Methods

### 2.1. Materials

FCC was supplied by BP Oil España S.A.U (Grao de Castellón, Spain) and CSW by Ideal Standard S.L.U (Valencia, Spain). FCC is the catalyst used after the cracking process, and CSW is material that derives from rejected units. Both materials must undergo a conditioning process to obtain the required fineness to improve their reactivity. FCC was milled in an industrial mill by Omya Clariana S.A (Tarragona, Spain). CSW units were firstly broken manually with a hammer and then placed inside a jab crusher (Restch BB200 model, Germany) to obtain a particle size less than 2 mm. Then the crushed CSW was milled in a jar roller mill for 6 h (Gabbrielli Roller 1 model, Calenzano, Italy)

Alkaline solutions were prepared by two methods: commercial reagents and an alternative silica source. The dissolution based on commercial reagents employed NaOH pellets of 98% purity (Panreac Quimica S.L.U, (Barcelona Spain) and Na_2_SiO_3_ (28% SiO_2_, 8% Na_2_O, 64% H_2_O) supplied by Merck S.L.U. (Barcelona, Spain) The dissolutions based on the alternative silica source were prepared, using a thermal bottle to dissolve RHA or DB with NaOH and H_2_O for 24 h. RHA was supplied by Dacsa S.A (Tavernes Blanques, Spain) and was milled in an industrial mill for 4 h. DB was supplied by Heineken España (Quart de Poblet, Spain) and was oven-dried at 105 °C for 24 h to remove moisture.

The chemical compositions of the waste materials are shown in Table 1. The equipment employed to analyze the composition was a Philips Magic Pro Spectrometer by the X-Ray fluorescence (XRF) Technique. As we can see in Table 1, RHA and DB had a higher percentage of silica. In both cases, the quantity of this oxide was employed to calculate its dose during the alternative silicate preparation for pastes and mortars.

**Figure 1:**
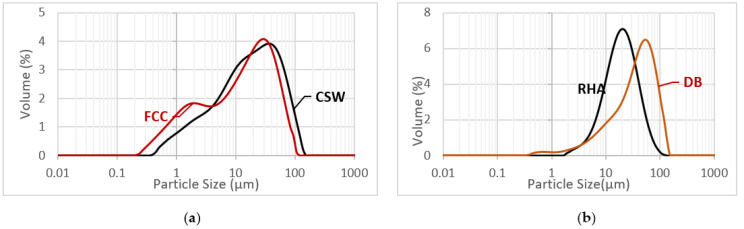
Particle size distribution curves by laser granulometry technique: (**a**) FCC and CSW; (**b**) RHA and DB.

The mean particle diameter of the particles of FCC, CSW, RHA, and DB was 17.12 µm, 31.24 µm, 20.30 µm, and 46.40 µm, respectively. These values were obtained by a laser dispersion analysis in a Mastersizer 2000 (Malvern Instruments, Malvern, U.K.). In Figure 1a,b the particle size distribution curves of starting materials are shown.

**Figure 2:**
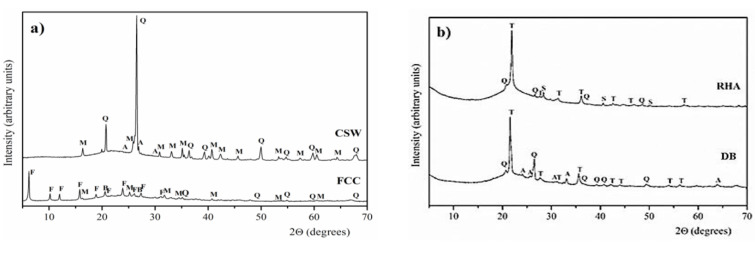
X-ray diffractograms of raw materials: (**a**) FCC, CSW; (**b**) RHA, DB (Q: quartz, M: mullite, A: anortite, B: albite, F: faujasite, T: cristobalite, S: silvine).

The XRD spectra of the waste materials are represented in Figure 2. The equipment employed was a Brucker AXS D8 Advance (Billerica, Germany). XRD spectra were measured from 10° to 70° 2θ at 20 mA and 40 kV with an angle step of 0.02°. The accumulation time in this step was 2 seconds. CSW presented as crystalline phases: quartz (Q, SiO_2_, PDFcard 331161), mullite (M, Al_6_Si_2_O_13_, PDFcard 150776) and anorthite (A, CaAl_2_Si_2_O_8_, PDFcard 411486). FCC also presented quartz and mullite, and it contained the presence of albite (B, NaAlSi_3_O_8_, PDFcard 200554) and faujasite (F, Na_2_Al_2_Si_4_O_12_.8H_2_O, PDFcard 391380). The main crystalline peaks of RHA and DB were associated with silica, and peaks were quartz and cristobalite (T, SiO_2_, PDFcard 391425). The presence of anorthite was observed in DB, and silvine (S, KCl, PDFcard 411476) was noted in RHA.

### 2.2. Experimental Procedure

As mentioned before in the Introduction, in this research an already published work was taken as a reference [34]. In the activator, the molar SiO_2_/Na_2_O ratio was 1.16 and the proportion of Na_2_O was 1.69 mol per kg of precursor. The precursor was the sum of CSW and FCC. The water/binder and sand/binder ratios were 0.45 and 3, respectively. The employed siliceous sand had a fineness modulus of 4.1 and a humidity percentage below 0.1%. To enhance CSW reactivity, a calcium source [35] (4% Ca(OH)_2_ in relation to the mass of the precursor) was added to the studied mixes. The same dose was employed to prepare pastes, but without sand.

Table 2 summarizes the dose and nomenclature for the analyzed pastes and mortars. Two CSW/FCC proportions were studied: (i) 70% CSW with 30% FCC (7-3 system); (ii) 50% CSW with 50% FCC (5-5 system). For each mixture of precursors, the alkaline dissolution was prepared with commercial (C) and alternative components: RHA + NaOH (R) and DB + NaOH (D). The symbol “m” represents the mortar specimens, while symbol “p” denotes paste ones.

The materials were cured under two conditions: in a thermal bath at 65 °C and 100% relative humidity for 7 days (mortars were demolded after 6 h and then cured for up 7 days); at room temperature (20 °C and 95% relative humidity) for 28 and 90 days.

Compressive strength was measured in specimens (4 × 4 × 16 cm^3^) according to Standard UNE-EN 196-1 [36]. Pastes were analyzed by different techniques (TG, XRD, FESEM). Thermogravimetric analyses were performed in a Mettler Toledo TGA 850 thermobalance (Germany) using a nitrogen atmosphere and a flow gas of 75 mL/min. The used crucibles were aluminum with pinholed lids. The analysis heat range went from 35 °C to 600 °C at a heating rate of 10 °C/min. The equipment utilized for the XRD analysis is previously described. Pastes were carbon-coated to characterize the reaction products by FESEM using FESEM ULTRA 55 (Zeiss, Jena, Germany).

## 3. Results and Discussion

### 3.1. Compressive Strength

**Figure 3:**
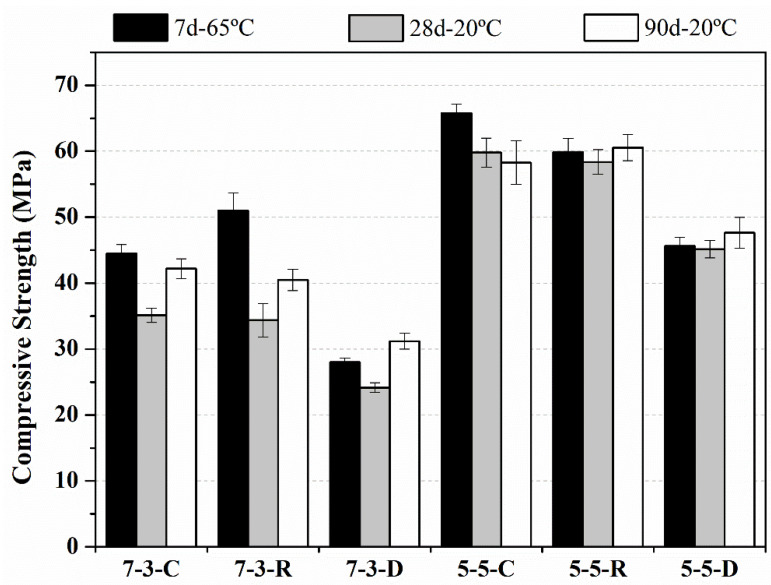
Compressive strength of the mortars cured at 65 °C for 7 days and cured at 20 °C for 28 and 90 days.

The compressive strengths of the mortars cured under the different assessed conditions are shown in Figure 3. We can see that the mortars with DB as a silica source (7-3-D and 5-5-D) had lower compressive strength than the mortars activated with both commercial reagents and the RHA-based activator. These differences in the mortars activated with RHA and those with DB can be attributed to the fineness of both silica sources: the mean particle size of DB was more than double that of RHA (40.60 vs. 20.30 µm). The dissolution rate of silica in the NaOH medium depends on the particle size of the employed silica source, and the amount of silicate in the DB-based activator was probably lower than that of the RHA-based activator.

When DB was employed as a silica source, mortars yielded different behaviors when comparing the precursor proportions (5-5 and 7-3) and curing treatments. For the 5-5-D sample, compressive strength was similar for the different applied curing treatments, with values between 45.2 MPa and 47.6 MPa. For the 7-3-D sample, the compressive strength values were lower than those obtained for the 5-5-D sample: this behavior can be attributed to the greater reactivity of FCC as a precursor compared to CSW. Similar values between curing ages for the 7-3-D series were obtained, and ranged between 24.2 MPa and 31.2 MPa. This was the worst series of mortars, but the compressive strength values were clearly higher than those of the mortars with only CSW as a precursor and prepared under the same alkaline conditions as the commercial reagents [9]. The compressive strength values of the mortars with only CSW came close to 9 MPa for 7 days curing at 65°C, but were only 3.7 MPa for the mortars cured for 90 days at 20 °C. The mortars with only FCC as a precursor obtained values of 45.3, 61.1, and 68.5 MPa for 7, 28, and 90 days, respectively. The only mortar with DB that yielded comparable values for the mortar with FCC and commercial reagents was mortar 5-5-D cured for 7 days at 65 °C [34].

The mixtures of the 5-5 type activated with commercial reagents (5-5-C) and with RHA (5-5-R) obtained the highest compressive strength values, either close to or higher than 60 MPa for all the studied curing conditions. These values are very positive from the practical and environmental point of views because these mixtures contained 50% CSW, which is a very high reuse CSW percentage. This implies marked CWS valorization, which has hardly been studied to date.

The behavior of the mixtures 7-3 cured at 65 °C differ compared to the commercial source and RHA activation: the compressive strength of the 7-3-R mortar was 51 MPa and the 7-3-C mortar yielded 44.5 MPa. For the room temperature curing conditions, the two mortar types yielded a similar compressive strength behavior, with values close to 35 MPa after 28 curing days and 40 MPa after 90 curing days, respectively.

The results obtained in the present research differ from those included in other reports. For example, Mejía et al. [21] obtained similar results for both pastes using DB and RHA, and compressive strength values were clearly lower than those of the paste activated with commercial reagents. They employed a mixture of 70% FA and 30% MK. The samples with commercial reagents yielded compressive strength values close to 75 MPa after 360 curing days. The samples with DB and RHA gave 38 and 36 MPa, respectively, at the same curing time, but the liquid/solid ratios of mixtures differed from commercial pastes and the alternative silicate pastes: 0.3 for the paste with commercial silicate, and 0.45 for the pastes with RHA and DB. These results, compared to those herein obtained, were significantly lower in compressive strength terms. Such behavior can be attributed to differences in the activator’s preparation procedure: Mejía et al. [21] only stirred RHA + NaOH for 24 h at room temperature, whereas our silica dissolution process of RHA was more intensive as the heat dissolution of NaOH increased the temperature of water/RHA or water/DE. We maintained this heat for several hours because the suspension was hermetically stored in a thermal bottle, a process that favors the dissolution of silica from RHA or DB. The work reported by Mejia et al. described how DB was calcined for 3 h at 400°C. This treatment can explain the similar behavior of diatomite in relation to RHA. Bagci et al. [22] calcined a natural diatomite. Their compressive strength values in the mixtures with MK of the samples with diatomite were higher than the mixtures activated with silica fume, 71 MPa versus 54 MPa, under the combined curing conditions (24 h at 50 °C and 6 days at room temperature). In the present study, only DB was dried, but not calcined. Calcination is a procedure that probably increases DB solubility.

### 3.2. Thermogravimetric Analysis

**Figure 4:**
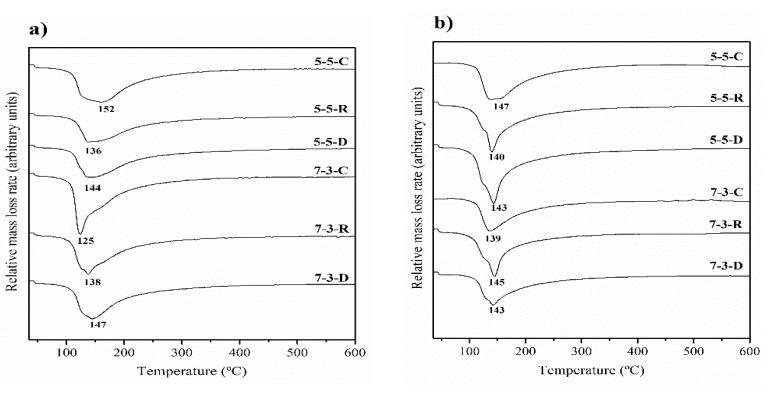
DTG curves: (**a**) cured for 7 days at 65 °C; (**b**) cured for 28 days at 20 °C.

Thermogravimetric analyses were performed for all the pastes under two different curing treatments: 7 days at 65 °C and 28 days at room temperature. The derivative thermogravimetric curves (DTG) are depicted in Figure 4. For all the analyzed pastes, the DTG curves presented a peak, which appeared at different temperatures depending on the activator and the proportion of precursors used in the formulation. After 7 days at 65 °C, when commercial reagents were employed as an activator (C), the peak centered at 125 °C for paste 7-3 and at 152 °C for paste 5-5. Within the interval of temperatures at which the peak appeared, different gels (N-A-S-H and N-(C)-A-S-H) lost their combined water [37,38,39].

Table 3 summarizes the total mass lost between 35 °C and 600 °C for the studied pastes. Generally, the highest mass loss was for the paste with the commercial activator. This phenomenon is typical when an alternative activator is employed; e.g., Moraes et. al. [40] compared sugar cane straw ash and commercial sodium silicate. They observed that the paste with the alternative activator had the lower mass loss, but concluded that the formed products were similar. In general, the mass loss at 28 days/20 °C was lower than that at 7 days/65 °C, which might be due to the different reaction kinetics for the two selected temperatures.

### 3.3. XRD Analysis

**Figure 5:**
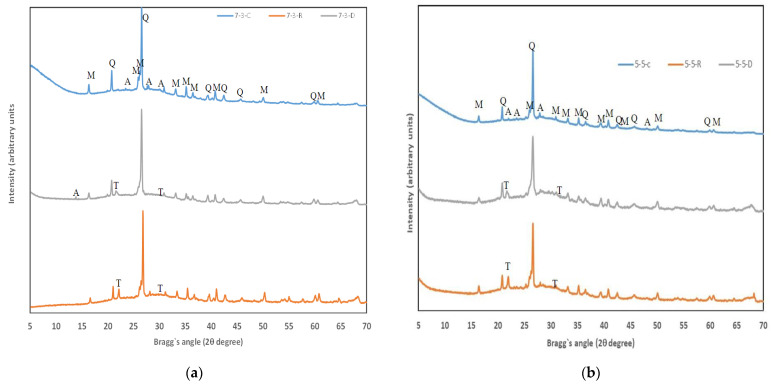
XRD diffractograms of the pastes cured for 28 days at 20 °C: (**a**) 7-3 type; (**b**) 5-5 type.

Figure 5 represents the XRD diffractograms of the pastes cured for 28 days. The crystalline compounds presented in the precursors were detected in all the pastes (Q: quartz, A: albite, M: mullite). In the pastes activated with DB and RHA, the presence of cristobalite (T) was also noted, a mineral present at a low proportion in the silica source. These crystalline phases (Q, A, M, T) were nearly inert to geopolymerisation reactions and remained after the curing process.

It should be noted that the peaks related to the presence of faujasite, a zeolitic phase which is the main crystalline compound present in FCC (Figure 1a), were not detected in paste types 7-3 or 5-5. This fact has been previously reported [41,42] and described how zeolite was dissolved with the activating solution to provide Al and Si for the geopolymerisation reaction and the formation of cementitious gel. It is noteworthy that in all the diffractograms, a marked deviation from the baseline took place between 15–40° 2θ. This can be attributed to the presence of the N-A-S-H and N-(C)-A-S-H gels formed during the geopolymerisation process [41,43]. Raw materials (CSW, FFC, RHA, DB) presented a deviation (hump or diffuse halo peak) at the baseline (see Figure 1) within the 15–30° range, and the maximum of the hump fell within the 21–24° range. After geopolymerisation, the hump shifted to higher 2θ values, within the 17–40° range, which meant that cementing gels formed from the dissolution of amorphous phases in raw materials [44]. After the reaction, the maximum of the hump in pastes also shifted to higher 2θ values (26–30°), which indicates the partial dissolution of amorphous phases in raw materials and the yielding of new amorphous phases. The added portlandite was not detected for all the pastes, which suggests that calcium was incorporated into reaction products. Reig et al. verified this fact by following a similar process with porcelain stoneware tiles and CSW activations [45,46].

### 3.4. FESEM Analysis

**Figure 6:**
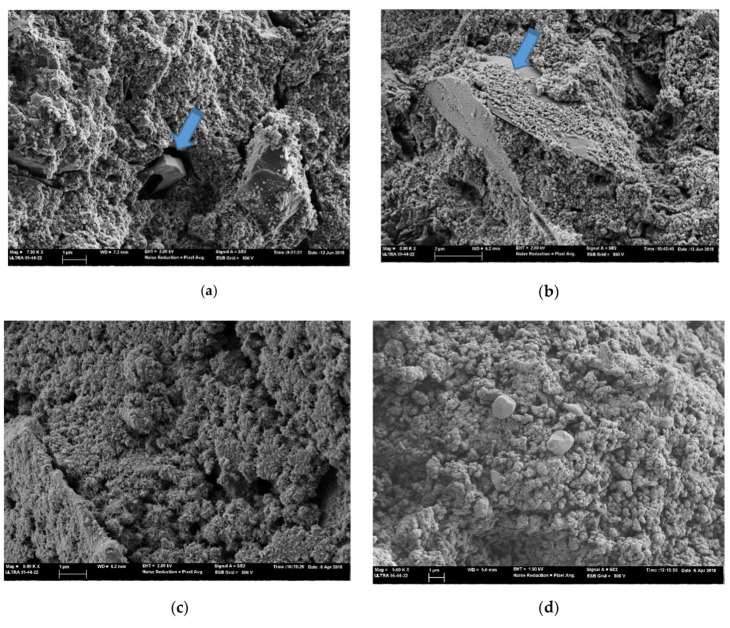

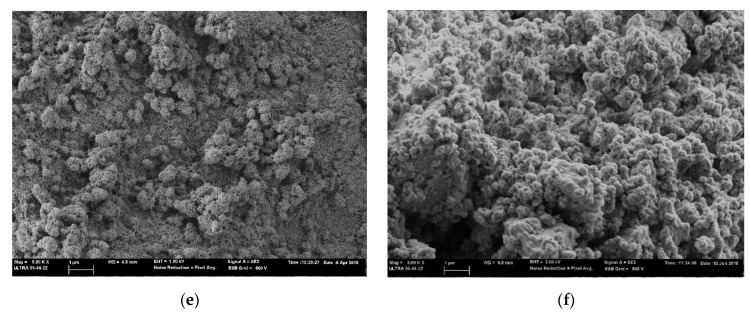
FESEM micrographs for the 5-5 type pastes cured at 65 °C/7 days and 20 °C/28 days. Arrows indicate unreacted CSW particles. (**a**) 5-5 C paste cured 7d 65 °C; (**b**) 5-5-C paste cured 28d 20 °C, (**c**) 5-5-R paste cured 7d 65 °C, (**d**) 5-5-R paste cured 28d 20 °C, (**e**) 5-5-D paste cured 7d 65 °C, (**f**) 5-5-D paste cured 28d 20 °C.

In Figure 6, FESEM micrographs are shown for pastes 5-5 cured for 7 days at 65 °C and 28 days at 25 °C, prepared using commercial sodium silicate (Figure 6a,b) as and NaOH activator, plus RHA (Figure 6c,d) and NaOH plus DB (Figure 6e,f). In all cases, a dense compact microstructure is observed, even though the pastes cured for 28 days were more compact than those cured at 65 °C. This would agree with the high strength found for the corresponding mortars.

**Figure 7:**
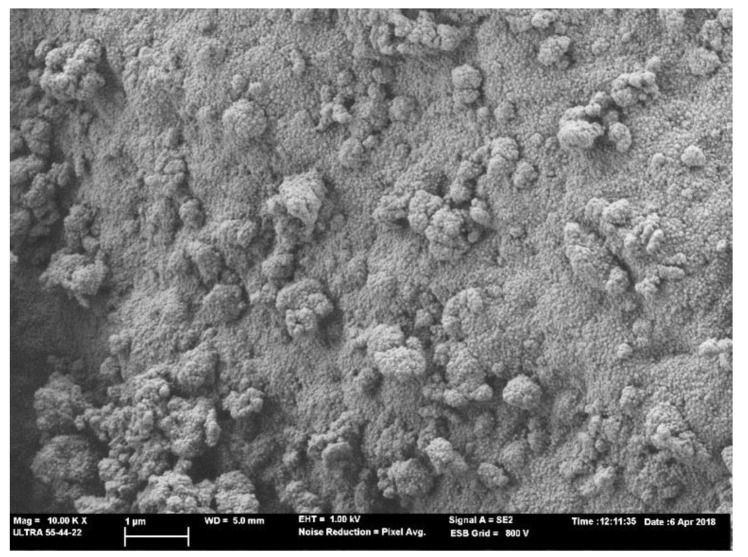
FESEM micrograph of the 5-5-R paste cured at 20 °C for 28 days: detailed view of the cementing gel.

In the pastes with commercial silicate, unreacted CSW particles were observed and particles were partially covered by the reaction products (Figure 6a,b). The principal products were N-A-S-H and N/C)A-S-H gels. These gel products have been previously detected [9]. Similarly in the mixtures with MK, which is a similar precursor to FCC, and using RHA as the silica source, the presence of this gel type has been reported by Kamseu et al. [16]. This gel was formed by the agglomeration of nanoparticles (detailed in Figure 7).

## 4. Conclusions

The main conclusions of this research work are summarized below:

- The mortars activated with RHA were comparable to the behavior of the mixtures activated with commercial reagents. The best option for reaching high compressive strength was to use the precursor with the 5-5 FCC/CSW ratio.
- Activation with DB obtained lower compressive strength values than the other matrices, but this strength sufficed for applications, which require strength below 50 MPa for the mixtures with the 5-5 ratio of precursors, and around 30 MPa with the 7-3 ratio.
- The main reaction product was a mixed gel, N-A-S-H and N-(C)-A-S-H, as corroborated by the TG, XRD, and FESEM analyses.

We generally conclude that the CSW/FCC binary mixtures can be an alternative as a precursor for preparing alkali-activated materials, and they obtained good mechanical strength results.

The possibility of using residual materials rich in silica, such as RHA and DB as part of the activator in alkali activated mixtures, was also demonstrated with similar mechanical strength and microstructure results to those obtained with commercial sodium silicate. This proposal contributes to materials sustainability.

## Figures and Tables

**Table 1:** Chemical composition of FCC, CSW, RHA, and DB (wt%).

	Al_2_O_3_	SiO_2_	CaO	Fe_2_O_3_	K_2_O	Na_2_O	P_2_O_5_	Other	LOI *
FCC	49.26	47.76	0.11	0.60	0.02	0.31	0.01	1.42	0.51
CSW	23.60	66.00	1.20	1.30	2.80	2.40	0.50	2.00	0.20
RHA	0.25	85.58	1.83	0.21	3.39	_	0.67	1.08	6.99
DB	5.67	81.70	1.28	3.71	0.86	1.30	0.36	1.78	3.34

* Loss of ignition.

**Table 2:** Dose of mortars and pastes.

	CSW (g)	FCC (g)	Na_2_SiO_3_ (g)	NaOH (g)	H_2_O (g)	RHA (g)	DB (g)	Ca(OH)_2_ (g)	Sand (g)
m-7-3-C	315.0	135.0	189.8	41.2	81.0	_	_	18.0	1350.0
m-7-3-R	315.0	135.0	_	60.8	202.5	62.1	_	18.0	1350.0
m-7-3-D	315.0	135.0	_	60.8	202.5	_	65.1	18.0	1350.0
m-5-5-C	225.0	225.0	189.8	41.2	81.0	_	_	18.0	1350.0
m-5-5-R	225.0	225.0	_	60.8	202.5	62.1	_	18.0	1350.0
m-5-5-D	225.0	225.0	_	60.8	202.5	_	65.1	18.0	1350.0
p-7-3-C	14.0	6.0	8.4	1.8	3.6	_	_	0.8	_
p-7-3-R	14.0	6.0	_	2.7	9.0	2.8	_	0.8	_
p-7-3-D	14.0	6.0	_	2.7	9.0	_	2.9	0.8	_
p-5-5-C	10.0	10.0	8.4	1.8	3.6	_	_	0.8	_
p-5-5-R	10.0	10.0	_	2.7	9.0	2.8	_	0.8	_
p-5-5-D	10.0	10.0	_	2.7	9.0	_	2.9	0.8	_

**Table 3:** Thermogravimetric mass loss (as %) of pastes, between 35°C and 600 °C.

Paste	7 d, 65 °C	28 d, 20 °C
7-3-C	11.95	8.77
7-3-R	9.61	8.03
7-3-D	9.72	7.44
5-5-C	11.24	10.23
5-5-R	8.55	9.00
5-5-D	9.83	10.77

## Data Availability

The data presented in this study are available on request from the corresponding author.

## Acronyms

FCC	Fluid catalytic cracking residue
CSW	Ceramic sanitary ware
RHA	Rice husk ash
DB	Diatomaceous waste from beer filtration
SDG	Sustainable Development Goals
N-A-S-H	Hydrated sodium aluminosilicate gel
FESEM	Field emission scanning electron microscopy
N(C)ASH	Hydrated sodium(calcium)aluminosilicate gel
PC	Portland cement
DE	Diatomaceaous earth (bibliography)
FA	Fly ash
MK	Metakaolin
XRF	X-Ray fluorescence
XRD	X-Ray Diffraction
DTG	Derivative thermogravimetric curve
